# The Impact of Assessing Patients' Sense of Security on Nurses' Intent to Report Safety Events: A Factorial Survey Experiment

**DOI:** 10.1111/jan.70483

**Published:** 2026-01-16

**Authors:** Patricia S. Groves, Yelena Perkhounkova, Maria Hein, Peter James Abad

**Affiliations:** ^1^ College of Nursing University of Iowa Iowa City Iowa USA; ^2^ College of Nursing University of the Philippines Manila Manila Philippines

**Keywords:** bias, factorial survey experiment, hospitals, nurses, patient safety, quantitative methods, safety reporting, vignette

## Abstract

**Aims:**

To evaluate whether information about patients' poor sense of security in hypothetical vignette scenarios increases nurses' projected intent to report safety events.

**Design:**

Quantitative, cross‐sectional factorial survey vignette experiment administered online.

**Methods:**

A convenience sample of 60 nurses from adult inpatient hospital units at a Midwest academic medical center participated in February 2025. Participants responded to demographic questions and eight factorial vignettes, each describing a patient‐reported safety breach and incorporating four patient‐related factors. Four vignettes included information that the patient had a poor sense of security, and four did not, presented in random order. Following each vignette, participants rated their level of concern about the patient's report, perceived harm to the patient, and likelihood of reporting the patient's concern. A linear mixed‐effects modelling approach, accounting for clustering within participants, was used to estimate the effects of the sense of security information factor on nurses' responses.

**Results:**

The sense of security information was associated with higher ratings of (a) degree of concern, (b) perceived harm to the patient, and (c) intent to report the patient's concern, after adjusting for vignette‐ and participant‐level covariates. The vignette patient's perception of physical harm was positively associated with all three ratings. Nurses' greater hospital experience was associated with lower ratings across outcomes.

**Conclusion:**

Obtaining information that the patient felt insecure was associated with heightened concern about the safety event, greater perceived harm, and increased intent to report the concern.

**Implications for the Profession and/or Patient Care:**

Sense of security assessment may be a risk‐agnostic, patient‐centered intervention that nurses can routinely perform, regardless of the safety event circumstances.

**Impact:**

Although a system of evidence‐based practices within a safety culture is essential to hospital safety efforts, nurses' judgements of and responses to patient safety concerns play a critical role and should not be overlooked.

**Reporting Method:**

STROBE guidelines.

**Patient or Public Contribution:**

This study did not include patient or public involvement in its design, conduct, or reporting.

## What Does This Paper Contribute to the Wider Global Clinical Community?

- This paper suggests a nursing intervention that centers on how the patient feels about their safety and can be used in a variety of patient encounters.

## 1 Introduction

Nursing bias affects patient safety practices in hospitals, resulting in safety disparities. Bias is generally defined as “the negative evaluation of one group and its members relative to another” (Blair et al. 2011, 71) and can influence care, which could impact patient safety outcomes. In one meta‐analysis, the pooled prevalence of overall patient harm in hospitals globally was 10% (Panagioti et al. 2019). NIH‐defined health disparity populations are at greater risk for poor safety outcomes (Agency for Healthcare Research and Quality [AHRQ] 2024). The problem is that nurses, like other clinicians, may be biased against certain patient characteristics within these populations (Thirsk et al. 2022), and implicit biases may shape nurses' judgements and subsequent intentions to act, further contributing to patient safety disparities (Groves, Farag, et al. 2025). As part of their routine work, nurses constantly and often unconsciously act to keep patients safe (Groves et al. 2017). However, nurses are commonly under time pressure, which makes implicit bias more likely to come into play (Thirsk et al. 2022). With nurses being the professionals spending the most time caring for patients at the bedside, it is critical to find ways to mitigate nursing bias in relation to the wide range of safety risks nurses encounter (Mitchell et al. 2023).

## 2 Background

Hospitalised patients experience threats to their safety and preventable harm. *Patient safety* is often defined as “freedom from accidental or preventable injuries produced by medical care;” those injuries are termed *adverse events* (AHRQ, n.d.). Globally, a meta‐analysis of hospitalised patients indicates an adverse event rate of 10%; of those, half are preventable and over 7% result in death (Schwendimann et al. 2018). AHRQ (2024) reported that following the COVID‐19 pandemic, many hospital safety indicators point to a decline in patient safety. Literature indicates increased risk of poor safety outcomes in publicly insured and racially minoritized hospital patients (Gangopadhyaya et al. 2023), with rural residents and non‐Hispanic Black individuals experiencing poorer outcomes (AHRQ 2024). Because patients report being unsafe or being harmed in ways clinicians don't see (Jeffs et al. 2022), the patient perspective on their own safety is critical.

However, system and interpersonal barriers may inhibit hospitalised patients from expressing their own safety concerns, thus preventing the hospital from developing a thorough and complete comprehension of organisational patient safety to take appropriate action (National Steering Committee for Patient Safety 2020). Patients are often unaware of, or don't know how to access, reporting mechanisms, including those meant for their own use; therefore, they report their concerns to their clinicians (Terry et al. 2022).

Unsurprisingly, the attitudes and encouragement of nurses towards patients voicing safety concerns are considered especially crucial (Vaismoradi et al. 2015). However, studies have shown that patients worry about clinician bias when voicing their safety concerns (Terry et al. 2022) for good reason, as nurses, like other clinicians, may demonstrate implicit bias in relation to patient attributes. *Implicit bias* is an unconscious mental association, influencing judgement and action without volition or even recognition (Greenwald et al. 2022). Healthcare professionals, including nurses, have levels of implicit racial and ethnic bias comparable to the general population (FitzGerald and Hurst 2017). Nurse bias may relate to attributes such as race, ethnicity, gender, and socioeconomic status (Thirsk et al. 2022).

Incident reports are “a key source of safety intelligence for hospitals” (Leon et al. 2024, 1). The patient's bedside nurse reports the vast majority of safety occurrences through the organisational incident reporting system (OIRS), which is designed to permit organisational learning and action (Goekcimen et al. 2023). However, moving from patient expression of concern to a nurse reporting the concern to the OIRS involves several nurse judgements, and is thus a process that may involve implicit bias. A nurse's bias may influence their judgement of a patient‐expressed safety concern, consequently resulting in systematic underreporting of concerns of patients who have historically been socially and economically marginalised (Schulson et al. 2021). Underreporting is a particular problem, because to improve care in the future, the organisation must learn from the present.

### 2.1 Current Approaches

Despite acknowledgment of the importance of nursing bias and attitudes towards patients voicing safety concerns, Thirsk et al. (2022) scoping review examining bias in nurses' judgement and decision‐making revealed only two research reports that tested interventions to mitigate nurse bias during nursing practice decision‐making. Neither was related to safety incident reporting. Of these studies, one tested the effect of pattern recognition‐based simulation on students' clinical decision making surrounding myocardial infarction (Walsh 2010), and, notably, one tested the effect of empathy‐inducing, perspective‐taking interventions on nursing students and nurses to reduce pain treatment bias (Drwecki et al. 2011).

Research focused on nurse incident reporting is largely focused on identifying perspectives, attitudes, and barriers to reporting rather than testing interventions (Smit and Peddle 2025). Many are specific to a certain type of event, such as a medication error, or to a certain level of event, such as near misses, and focus on nurse‐involved or ‐observed events. On the whole, there is a lack of focus on how patients' expressed safety concerns are made sense of by the nurse and subsequently (un)reported, and a corresponding lack of focus on intervening at this point.

### 2.2 A New Approach

Considering this, together with colleagues we previously evaluated the role bias plays in nurses' judgements of credibility (believability and convincingness) and importance (degree of concern) of a patient‐expressed safety concern as well as their intent to report it via the OIRS. Furthermore, we evaluated to what degree nurse judgements of credibility and importance mediated the relationship between patient characteristics and the nurses' intent to report the patients' safety concern. We found that patient characteristics, such as socioeconomic status (SES), race and ethnicity, and gender, as well as patient communication style, type of safety event (medication or miscommunication), and whether the patient had a perception of harm, directly or through interactions impacted judgement of credibility, judgement of importance, and/or intent to report (Groves, Farag, et al. 2025). We further determined that the nurses' judgement of importance had a direct effect on the nurses' intent to report and mediated the relationship between patient SES and nurse intent to report as well as between race and nurse's intent to report. However, nurses' judgement of the credibility of the patients' report had no significant effect on intent to report, nor did it mediate relationships between the patient characteristics and the nurses' intent to report (Groves, Perkhounkova, et al. 2025).

The current study is premised on two important implications from this previous work. First, the predominant approach of nurse education and training, which is based on reporting specific types of harm (e.g., medication error or near miss), while valuable, does not address the interplay of factors and complexity of relationships found in our earlier work. This suggests that “mitigation should also focus on a complex system where bias is an inevitable and generally invisible part of humans providing care” (Groves, Farag, et al. 2025, 504). Second, despite an assumption that nurse bias reveals itself in judgement of lesser credibility, credibility did not appear to be a mediating factor between patient characteristics and the nurses' intent to report. The results instead suggested that interventions should focus on the nurse's judgement of patient report importance as a more promising area of investigation and intervention. We therefore proposed an intervention of enabling perspective‐taking through assessing the patient's perspective on their safety (i.e., sense of security), supporting the need to both mitigate inevitable bias and focus on the judgement of importance made by nurses when evaluating a patient‐expressed safety concern.

Safety as defined by researchers and healthcare professionals generally focuses on a lack of iatrogenic injury, particularly preventing observable injuries that result in achieving regulatory safety metrics (Groves et al. 2023). However, to patients, their perception of safety—or sense of security (SoS)—may be more pertinent. A decreased sense of security indicates the patient feels vulnerable to potential harm, whether it be physical, emotional, or psychological (Lovink et al. 2015; Mollon 2014). Consequences of decreased sense of security include increased suffering and distress, and may include loss of hope, frustration, fear and anxiety, increased vigilance, paranoia, and mental exhaustion (Dabaghi et al. 2020; Groves et al. 2023; Hupcey 2000; Mollon 2014). These harms add to any consequences from an (un)observable physical injury. To focus on SoS, Mollon (2014) encourages nurses to take the perspective of the patient—whose sense of security can vary on an hourly or daily basis—by simply asking them if they feel safe.

## 3 The Study

The purpose of the current study was to evaluate a potential intervention intended to mitigate nurses' biases regarding patient‐expressed safety concerns, with the intention of improving patient safety in hospitals. Specifically, our primary aim was to evaluate whether information about patients' poor sense of security (SoS) in hypothetical vignette scenarios would increase nurses' projected intent to report safety events. We hypothesised that information indicating that the patient felt insecure would increase nurses' ratings of intent to report. Because vignette factors were experimentally manipulated and randomly assigned, we interpret the participant responses as being elicited by the manipulated factors within the hypothetical scenarios presented in the survey. We were also interested in the impact of the SoS factor on nurses' judgement of importance (i.e., concern about patients' report), which was the theorised intervention target, and the impact on the nurses' perception of harm to the patient, to provide additional insight into how nurses may interpret a patient's report of poor SoS.

### 3.1 Sense of Security Information Factor

The SoS information factor (the patient responds to a SoS assessment by indicating insecurity) was designed to support the cognitive empathy states of perspective‐taking: the nurse imagining how they would feel and think in the patient's situation, and the nurse imagining how the patient feels and thinks (Batson and Ahmad 2009). Perspective‐taking has been demonstrated to increase (1) empathic concern towards the other, (2) positive attitudes towards the other, and (3) willingness to help the other (Batson and Ahmad 2009). For example, a perspective‐taking intervention has been demonstrated to lower the odds of pain treatment bias against Black patients and those with low SES (Hirsh et al. 2019). Enabling perspective‐taking through assessing the patient's perspective on their own safety was expected to support both (1) the need to mitigate inevitable bias and (2) focus on the judgement of importance made by nurses when evaluating a patient‐expressed safety concern. Incorporating the question “Do you feel safe?” into the nurse's evaluation of a patient‐expressed safety concern not only provides important information to the nurse, but it also functions as an easy, routine intervention that can be applied to a variety of patient interactions and complex situations.

## 4 Methods

### 4.1 Research Design and Overview

We performed a quantitative, cross‐sectional factorial survey experiment using vignettes in an electronic survey format. Factorial surveys combine a survey with a multifactorial experimental design, boosting internal validity through experimental control and randomisation (Auspurg and Hinz 2015). Factorial surveys are ideal for (a) assessing judgement, attitudes, and decision‐making, (b) studying issues where the context matters and multiple factors may impact participant response, (c) asking about sensitive or controversial topics where participants may exhibit social desirability bias, and (d) when setting up a controlled experiment in situ is impossible (Atzmüller and Steiner 2010; Auspurg and Hinz 2015; Aviram 2012; Ludwick et al. 2004; Taylor 2006). Factorial surveys thus allow for manipulation of multiple factors expected to influence judgements, attitudes, and decision‐making, which would be impossible to do in clinical settings.

### 4.2 Setting and Sample

We recruited nurses working on adult inpatient hospital units (including medical‐surgical, specialty acute care, intensive, and step‐down, excluding women's health) as bedside (staff) nurses at a Midwest US academic medical center during February of 2025. Based on the study budget, we recruited a total of 60 participants, yielding 480 vignette‐based observations (60 participants × 8 vignettes each). Using data from our earlier study (Groves, Farag, et al. 2025) and assuming an 8‐period crossover design with two treatments (intervention and control) and a two‐sided type I error rate (*α* = 0.05), we estimated that a sample of 60 participants would provide 95% power to detect a mean difference of 0.2 points for concern about patient report and 78% power for intent to report a patient safety concern. The mean difference of 0.2 points was considered clinically and practically meaningful by the study team for this exploratory study phase evaluating a potential SoS factor effect. The sample of 60 nurses, determined primarily by budgetary constraints, was expected to include a diverse range of education, work experience, and hospital units. However, most participants were anticipated to identify as female and White, and no attempt was made to balance participant characteristics. Our previous research indicated that responding to eight vignettes did not cause participant fatigue. Finally, the total of 480 observations (240 control and 240 intervention) allows robust estimation of intervention effects for a future study.

Participants were recruited through a combination of advertisements (e.g., flyers) within the institution on the units where eligible nurses worked and through those units' intra‐department communications (e.g., unit newsletters). Both methods included (1) an overview of the study with an explanation that we wanted to know more about how nurses respond to different patient concerns, (2) contact information for the PI, and (3) a link and QR code to the electronic survey. Upon following the link, potential participants answered a screening question and were given the opportunity to enrol. Nurses were compensated $25 for completion of the survey.

We continued recruitment until we had 60 complete survey responses. Surveys abandoned before completing all vignettes (*n* = 12), along with those in which participants reached the end of the survey but lacked responses to some vignette questions (*n* = 8), were excluded from the analysis.

### 4.3 Instruments

The survey was delivered and submitted through the web‐based interface, Qualtrics, accessible via both computers and mobile devices.

#### 4.3.1 Demographics

Participants identified the type of unit they worked in (adult acute care, adult intensive care or step‐down, adult mental health, or adult post‐acute care), and their gender, race and ethnicity, most advanced nursing degree, number of years as a nurse, number of total years working in a hospital, and age.

#### 4.3.2 Vignettes

In addition to demographic questions, the survey included eight vignettes that each described a safety breach as reported by a patient and incorporated four patient factors: ethnicity, gender, socioeconomic status (SES), and perception of physical harm. See Table 1 for operationalisation of factors in the vignettes. An additional factor of SoS assessment information vs. no SoS assessment information was included at the end of vignettes. Participants received four vignettes with the SoS information, and four vignettes without it. We adapted the vignette template from one we successfully tested and used in a previous factorial survey experiment study (Groves, Farag, et al. 2025). The vignette template was drafted to tell a short story encompassing the literature‐indicated factors while remaining realistic despite variation in the factor levels (Aviram 2012). The scenario was validated through extant literature and expert input in nursing, error reporting, factorial surveys, healthcare bias, clinical practice, patient advocacy, and diversity to ensure construct validity and realistic content across different clinical settings (Groves, Farag, et al. 2025).

**TABLE 1:** Operationalisation of vignette factors into vignettes.

Vignette factors	Operationalised levels	Location in vignette
Gender	Masculine or feminine pronouns	Story text, photo, photo caption
Ethnicity	Darker skin or lighter skin	Photo
Socioeconomic status	Can or can't afford	Story text
Patient‐expressed physical harm	Feel okay, feel awful	Story text

The SoS assessment information was incorporated into vignettes by appending the following text at the end of the vignette: “You ask the patient if they feel safe, and they say, ‘I was so scared [or worried]; I keep thinking it might happen again.’ You're unable to verify the patient's account through the medical record.” Vignettes without SoS assessment information simply ended with “You're unable to verify the patient's account through the medical record.”

After each vignette, participants responded to three questions, using the 11‐point visual analogue scales (Auspurg and Hinz 2015). The participant was asked to consider their responses as if they were the nurse interacting with the hospitalised patient. The questions were: (1) How concerned are you about what the patient has reported? (2) How likely do you think it is that this patient has been harmed? (3) How likely are you to communicate the patient's concern through your organisation's incident reporting system? Possible responses ranged from “1—Not at all” to “11—Extremely.” The first and third questions were identical to ones used in our previous work described above (Groves, Farag, et al. 2025; Groves, Perkhounkova, et al. 2025).

The full universe of 16 vignettes (2 × 2 × 2 × 2) was created from the 4 factors of 2 levels each, including (1) a short story wherein a patient reported a safety event and (2) an AI‐generated photo of the patient with a name caption (e.g., Mr. Jones or Ms. Nelson). The vignette text template was based on previously validated vignettes, but with fewer patient factors (Groves, Farag, et al. 2025). A version of each vignette with either the SoS information or no SoS information ending was created, using a unique photo and name for each of the 32 (16 × 2) vignettes.

**FIGURE 1:**
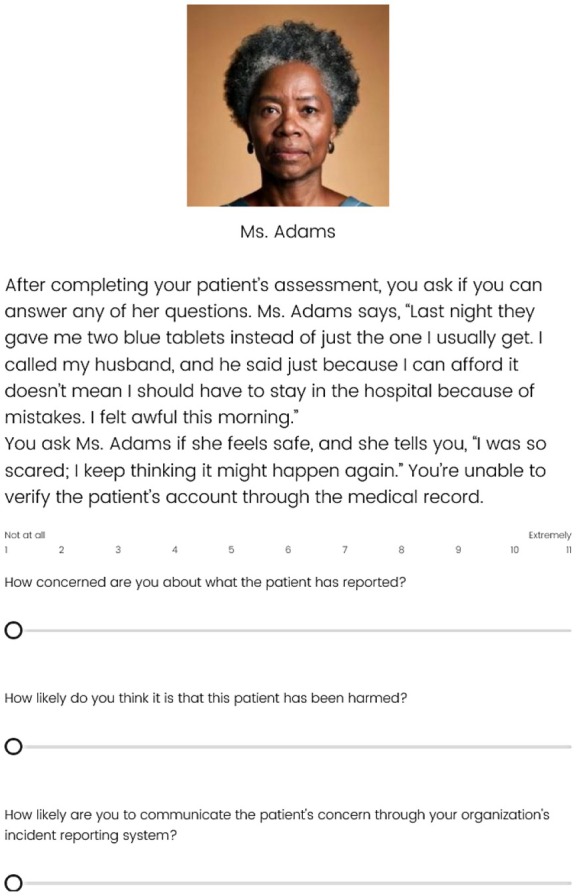
Example of a survey vignette with SoS assessment information ending.

The 32 vignettes were apportioned into 30 decks of eight vignettes so that each deck included a random selection of four SoS assessment information vignettes with equal representation of ethnicity and gender and four no‐SoS assessment information vignettes with equal representation of ethnicity and gender. Within the survey, each participant was assigned to one random deck of the 30 total decks, maintaining equal use of each deck within the total sample (each deck was used two times). Within the individual surveys, vignettes were presented in random order. The vignette template was as follows, with italicised words between brackets illustrating possible levels of each factor: “After completing your patient's assessment, you ask if you can answer any of [*her/his*] questions.” Your patient says, “Last night they gave me two white pills instead of just the one I usually get. I called my [*husband/wife*], and [*he/she*] said [*I can't afford/just because I can afford it doesn't mean I should have*] to stay in the hospital because of mistakes. [*I felt terrible this morning/I felt okay this morning, though*]. [*You're unable to verify the patient's account through the medical record./You ask the patient if they feel safe, and they say, “I was so scared/worried; I keep thinking it might happen again.” You're unable to verify the patient's account through the medical record*.]” See Figure 1 for an example of a vignette with the SoS information ending and subsequent questions for the participant.

#### 4.3.3 Validity and Reliability

We established internal validity by experimental control via random assignment of the independent variables (factors) via vignette blocks to participants (Auspurg and Hinz 2015). We used a vignette template previously content‐validated by clinical experts, a panel of patient advocates, and a minority academic diversity coordinator, but slightly simplified to include fewer patient characteristics (Groves, Farag, et al. 2025). Construct validity was supported by using factors suggested by prior research (Jasso 2006). Random order presentation of vignettes within the deck to decrease serial order dependency enhanced reliability, as did instructions previously tested for clarity to decrease measurement error (Auspurg and Hinz 2015; Steiner et al. 2016).

### 4.4 Data Collection

We collected survey data via the secure online Qualtrics application. After following the link, participants viewed (1) a welcome screen with the survey title, (2) a single required screening question to determine if they worked in an adult inpatient area of the hospital (not including women's health), (3) a consent screen approved by the IRB requiring agreement to proceed, (4) demographic questions, and (5) the series of eight vignettes with the three questions for each. On completion, participants had the option to be transferred to another unlinked survey to submit information needed to obtain compensation for participation. Collected survey data were imported to a secure database to run statistical analyses.

### 4.5 Ethical Considerations

The University of Iowa Institutional Review Board approved the study as exempt on December 31, 2024 (#20241181) and provided a waiver of written consent. Collected data were anonymous and automatically assigned a study ID. Participants entered their responses directly into the Qualtrics application using a device and location of their choice. All relevant physical and electronic methods of securing the data were used.

### 4.6 Data Analysis

SAS 9.4 software was used to organise and examine the data and to calculate descriptive statistics for all study variables, including the means and standard deviations for continuous variables and the frequencies and percentages for categorical variables. To address the study aims, we used a linear mixed modelling (LMM) approach, implemented with SAS procedure MIXED, to estimate the effects of the SoS assessment information on nurses' judgements. This approach is appropriate for the study's hierarchical data, as it accounts for the clustering of vignettes within participants by including a random effect. Model parameters were estimated with the residual maximum likelihood (REML) method. We examined residuals graphically to identify influential observations and violations to normality and homogeneity of variance assumptions (Cohen et al. 2014). No violations of assumptions or apparent outliers were found.

#### 4.6.1 Models for Nurse Judgements

Separate random intercept models were developed for each of the three dependent variables corresponding to survey questions, representing participants' ratings of (1) concern about patient report, (2) perceived harm to the patient, and (3) intent to report the patient concern. We estimated the fixed effect of the SoS assessment information factor while controlling for vignette‐level factors (Level 1) and participant‐level factors (Level 2). Level 1 factors included patient gender, ethnicity, socioeconomic status, and perception of harm, and Level 2 factors included participant characteristics such as nurse education, unit type, and nursing experience in the hospital. Nursing experience was centered at the mean years of experience. Participant gender and race/ethnicity were not included in the models due to sample homogeneity (90% females, 93% Whites). We tested interactions between all covariates and the SoS information factor to explore potential moderation effects on nurse judgements; no significant interactions were found. An unadjusted significance level of 0.05 was used to evaluate SoS information effects and the relevance of covariates. Intraclass correlation coefficients (ICCs) were calculated from the null (intercept‐only) models to estimate the proportion of variance in each dependent variable attributable to participants.

## 5 Results

### 5.1 Sample Characteristics

Demographic and occupational characteristics of the participants are presented in Table 2. Most participants identified as White (93.3%) and female (90.0%). A bachelor's degree in nursing was the most advanced degree earned by 63.3% of participants, while an additional 15.0% of participants had earned a master's degree. More participants reported working in an intensive care or a step‐down unit (73.3%) than in an acute care unit. Participants were about 33 years old on average (SD = 9.8), ranging from 22 to 64 years, with a mean of 8.5 years of nursing experience also reflecting a large range (SD = 8.7, range 0.5–37.0). Years worked in the hospital averaged 9.5 (SD = 7.9, range 0.5–37.0).

**TABLE 2:** Participant demographic and occupational characteristics.

Variable	Mean ± SD	Range
Number years worked as nurse	8.5 ± 8.7	0.5–37.0
Years total worked in a hospital^a^	9.5 ± 7.9	0.5–37.0
Participant age	32.9 ± 9.8	22.0–64.0

Variable	*n*	%
Gender		
Female	54	90.0
Male	6	10.0
Race/ethnicity		
White	56	93.3
Asian	2	3.3
Hispanic/Latino	1	1.7
Prefer not to answer	1	1.7
Most advanced degree		
Associate (ADN)	13	21.7
Bachelor (BSN)	38	63.3
Master (MSN)	9	15.0
Inpatient hospital unit type		
Acute care unit	16	26.7
Intensive care/step‐down unit	44	73.3

*Note:* *N* = 60. Percentages may not sum to 100% due to rounding.

^a^ *N* = 59.

### 5.2 Intercept‐Only Models for Nurse Judgements

Accounting only for clustering within participants, the estimated means and 95% confidence intervals for nurse ratings were: 6.85 (95% CI: 6.30 to 7.39) for concern about patient report, 4.88 (95% CI: 4.22 to 5.54) for perceived harm to the patient, and 5.96 (95% CI: 5.16 to 6.76) for intent to report patient concern. Intraclass correlation coefficients (ICCs) were calculated as 0.81, 0.84, and 0.92 for concern, perceived harm, and intent to report, respectively, indicating that most of the variation in these outcomes was attributable to differences among participants.

### 5.3 Final Models for Nurse Judgements

The final models are presented in Table 3. The SoS assessment information effect was statistically significant in all three models and was estimated at 0.42 points (*p* < 0.001) for nurse concern, 0.25 points (*p* = 0.009) for perceived harm to the patient, and 0.32 points (*p* < 0.001) for intent to report, reflecting adjustment for vignette‐level and participant‐level covariates. Because one participant did not provide years worked in the hospital, a significant covariate in all models, the final analyses were based on *N* = 59 nurses.

**TABLE 3:** Linear mixed models for nurses ratings related to a safety event.

Variables	Concern about patient report					Perceived harm to the patient					Intent to report patient concern				
	*b*	95% CI		*t*	*p*	*b*	95% CI		*t*	*p*	*b*	95% CI		*t*	*p*
Intercept	7.03	5.51	8.55	9.28	< 0.001	4.40	2.65	6.15	5.04	< 0.001	6.84	4.67	9.00	6.33	< 0.001
*Patient characteristics*															
Patient gender: female vs. male	0.09	−0.08	0.25	1.02	0.309	0.07	−0.12	0.26	0.74	0.459	0.12	−0.03	0.28	1.61	0.107
Patient ethnicity: dark skin vs. light skin	0.11	−0.06	0.28	1.27	0.205	**0.27**	**0.08**	**0.45**	**2.82**	**0.005**	0.15	0.00	0.30	1.92	0.056
Patient SES: lower vs. higher	0.00	−0.18	0.17	−0.05	0.963	−0.01	−0.20	0.19	−0.06	0.956	−0.07	−0.23	0.09	−0.89	0.372
Patient perception of harm: present vs. absent	**0.58**	**0.41**	**0.76**	**6.49**	**< 0.001**	**0.63**	**0.43**	**0.82**	**6.30**	**< 0.001**	**0.42**	**0.26**	**0.58**	**5.16**	**< 0.001**
**SoS information:** present vs. absent	**0.42**	**0.25**	**0.58**	**4.84**	**< 0.001**	**0.25**	**0.06**	**0.44**	**2.62**	**0.009**	**0.32**	**0.17**	**0.48**	**4.20**	**< 0.001**
*Participant characteristics*															
Years worked in a hospital^a^	**−0.08**	**−0.16**	**−0.01**	**−2.26**	**0.028**	**−0.14**	**−0.22**	**−0.05**	**−3.14**	**0.003**	**−0.15**	**−0.26**	**−0.04**	**−2.76**	**0.008**
Type of unit: Adult intensive care/step‐down vs. adult acute	−0.55	−1.89	0.78	−0.83	0.409	−1.28	−2.82	0.26	−1.67	0.101	−1.16	−3.07	0.75	−1.22	0.228
Advanced nursing degree															
BSN vs. ADN	−0.56	−1.94	0.82	−0.81	0.422	1.04	−0.55	2.63	1.31	0.195	−0.78	−2.76	1.20	−0.79	0.431
MSN vs. ADN	−0.07	−1.90	1.76	−0.08	0.936	0.88	−1.23	2.99	0.84	0.407	0.04	−2.58	2.66	0.03	0.976

*Note:* *N* = 59 nurse participants and 472 observations. b = unstandardised regression coefficient estimate; *p* = *p*‐values for a *t*‐test of significance of an effect or a level of an effect; bolded values are significant at *p* < 0.05.

Abbreviations: 95% CI, 95% confidence interval; ADN, Associate Degree in Nursing; BSN, Bachelor of Science in Nursing; MSN, Master of Science in Nursing; SES, socioeconomic status; SoS, sense of security.

^a^ Years worked in a hospital was centered.

#### 5.3.1 Vignette‐Level Factors

Among patient characteristics, patients' perception of harm was significant in all models, increasing ratings by an estimated 0.42 to 0.63 points (*p* < 0.001). Additionally, the patient's dark skin was associated with higher nurse ratings of perceived harm (*b* = 0.27, *p* = 0.005).

#### 5.3.2 Participant‐Level Factors

Among participant characteristics, additional years worked in the hospital were associated with lower ratings across all models: *b* = −0.08 (*p* = 0.028) for concern, *b* = −0.14 (*p* = 0.003) for perceived harm to the patient, and *b* = −0.15 (*p* = 0.008) for intent to report. Nurse education and unit type were not significantly associated with any of the judgements.

## 6 Discussion

We found that including within the vignette an SoS assessment with a response indicating the patient's feeling of insecurity elicited an increase in the ratings of all three dependent variables: degree of concern (judgement of importance), perceived harm to the patient, and the intent to report the patient concern to the OIRS. The vignette patient's perception of physical harm was also associated with increased ratings in the three models, regardless of the presence or absence of the SoS assessment information. A negative association was found between years of the participant's hospital experience and the three nurse ratings; additional years of hospital experience were associated with decreased intent to report, concern about patient report, and perceived harm to the patient. Other patient characteristics were not associated with nurse ratings, with one exception: patients' darker skin was associated with increased participants' perception of harm to the patient. The patient's gender and SES were not associated with any ratings, nor were participant hospital unit or nursing degree.

The SoS assessment information factor impacted the nurse ratings as expected, and in the expected direction. However, the SoS assessment information in this study included both asking the patient if they feel safe and a response from the patient that indicated a poor sense of security. Future work testing SoS assessment as a potential intervention should determine if it is (1) only the nurse asking the question or (2) both asking and the patient's response that impact the ratings. It is possible that just the act of asking the question is consequential, regardless of the patient response.

These results are also consistent with previous work identifying that the presence of harm is a criterion nurses use to determine if they should report an event to the OIRS (Groves et al. 2021). In this study, both the patient's expressed perception of physical harm and the patient's decreased SoS identified through the SoS assessment were associated with increased intent to report. As previously noted, it is important that future work differentiates between the effects of assessing SoS and the effects of the patient's response to the assessment.

It is interesting that years of hospital nursing experience were inversely related to nurses' ratings. Research in this area is limited; however, Pfeifer and colleagues surveyed paediatric nurses to assess intention to report safety events in relation to psychological safety and high reliability organisations (Pfeifer et al. 2023). The investigators used a modified version of the Frequency of Events Reported subscale of AHRQ's Hospital Survey on Patient Safety Questionnaire to measure safety event reporting intentions. Analysis indicated that with each additional year of practice experience, odds of achieving the maximum intent to report safety events score increased by a factor of 0.3. This is consistent with our previous work (Groves, Farag, et al. 2025), where we found that participants' years of experience were associated with increased participant concern and intent to report to the OIRS, in contradiction to the current study results. While our previous study did not test this SOS assessment information as a factor, it did involve more total vignette factors. Additionally, a much greater percentage of participants worked in intensive care and step‐down areas in this study than in the previous study (73.3% vs. 35.8%, respectively), participants were somewhat younger (mean age 33 vs. 39 years), had less experience working in a hospital (9.5 vs. 11.1 years), and were more homogeneous with respect to race and gender (Groves, Farag, et al. 2025). It is difficult to tell how any of those differences may have influenced this study's findings, but it suggests other unidentified variables of interest may play a role in nurses' decisions to report safety events. Future work might include examining the influences of years of nursing experience and unit safety culture on nursing perception of risk, harm, and OIRS use.

Also interesting is the heightened perception of harm for vignette patients with darker versus lighter skin. We were unable to locate published research reports relating nurses' harm perception to skin colour, race, or ethnicity, though studies have revealed different rates of reporting and types of events reported for patients of different racial or ethnic backgrounds (e.g., Thomas et al. 2020, 2021). The design of this study does not allow for interpretations of that relationship, but there are several possibilities. In our previous related study (Groves, Farag, et al. 2025), we noted that judgements influenced by race and ethnicity were multifactorial. Examining additional factors in concert with race and ethnicity, as well as interactions among different judgements, might expand our understanding of the relationship found in this study and have important implications for equity in safety event reporting in hospitals. Understanding variation in safety event reporting would provide opportunities for improving detection and organisational learning for these events across patient populations and contexts.

The primary strength of this study was its factorial survey experimental design, which enabled assessment of the dependent variables with inclusion of multiple vignette factors, while minimising the impact of social desirability bias, and allowing observation of phenomena otherwise unobservable in situ. The vignette factors were experimentally manipulated and randomly assigned so that we could determine if participant responses were elicited by the factors presented. However, survey vignettes, by their very nature, are over‐simplified representations, and cannot replicate the variety, complexity, and unpredictability that characterise actual clinical practice environments; thus, vignettes are insufficient for determining real‐life causality. There were other limitations, including the inability to differentiate between the effects of assessing SoS and the effects of the patient's response. A third limitation was a small number of factors incorporated into the vignettes. Fourthly, AI‐generated photos were created using instructions such as “create a photorealistic portrait of a white, middle‐aged woman with a neutral expression,” but they were not normed by external reviewers to determine if skin tone and gender were consistently interpreted the same way. Participants were notified at the end of the survey that all visuals were AI‐generated. A fifth limitation was that those who chose to respond to study advertisements may in some ways differ systematically or meaningfully from nurses who did not respond, reducing representativeness, generalisability, and introducing the potential for bias. Additionally, the study sample was drawn from a single facility within an academic health system, with a mostly white and female population, limiting generalisability to other geographic and hospital care delivery locations and more diverse populations.

Future research should incorporate more patient and event factors into the vignettes, allowing for more complex intersectionality and nuanced examination of relationships. Likewise, additional nursing intentions for action could be explored. Future studies should be designed to differentiate between the act of assessing SoS and the patient's response to the SoS assessment. Furthermore, testing of the SoS assessment information vignette factor and examination of relationships should take place within a larger sample representing a wider range of hospital organisations with more diverse nursing populations, in a greater geographic area. Further testing is needed to see if these findings are robust enough to be replicated in simulated or real‐world practice environments. Finally, the design of the current study does not allow identification of the cognitive, psychological, and/or social mechanisms driving the associations found in this study. There is a need to develop methodology (or to combine known methods such as simulation and cognitive interviewing in new ways) that allows for rigorous testing of safety communication interventions while also exploring the interacting cognitive and psychological processes and social environment influencing intervention success.

## 7 Conclusions

Assessing a patient's SoS and receiving a response indicating patient insecurity within the vignettes elicited intensified concern about the safety event (judgement of importance), heightened perception that the patient had been harmed (judgement of harm), and increased intent to report the patient concern to the OIRS. While SoS assessment requires further testing to determine its effectiveness and how best to apply it, this line of inquiry thus far suggests that SoS assessment may benefit nurses in clinical practice. Persuading the nurse to obtain information about whether the patient feels safe may also influence sensemaking on the part of the nurse, contributing information about “what is going on here” and thus influencing the response to “what do I do next?” (Groves et al. 2021). Regardless of the mechanism, SoS assessment may be a risk‐agnostic, patient‐centered intervention that can be routinely performed by a nurse regardless of the safety event circumstances. Further testing should be performed to evaluate this patient‐centered approach for influencing nursing judgement and prompting nursing action regarding patient‐expressed safety concerns.

## Author Contributions

All authors have agreed on the final version and meet at least one of the following criteria (recommended by the ICMJE*): (1) substantial contributions to conception and design, acquisition of data, or analysis and interpretation of data; (2) drafting the article or revising it critically for important intellectual content.

## Funding

This research received grant support from the University of Iowa College of Nursing.

## Disclosure

Statistics: Co‐author Yelena Perkhounkova is a statistician.

## Conflicts of Interest

The authors declare no conflicts of interest.

## Data Availability

The data that support the findings of this study are available from the corresponding author upon reasonable request.
